# Versatile Biodegradable Poly(ester amide)s Derived from α-Amino Acids for Vascular Tissue Engineering

**DOI:** 10.3390/ma3042346

**Published:** 2010-03-26

**Authors:** Pooneh Karimi, Amin S. Rizkalla, Kibret Mequanint

**Affiliations:** 1 Department of Chemical and Biochemical Engineering, The University of Western Ontario, London, ON, Canada; E-Mail: pkarimib@uwo.ca (P.K.); 2 Division of Biomaterials Science, Schulich School of Medicine and Dentistry, The University of Western Ontario, London, ON, Canada

**Keywords:** biodegradable polymers, α-amino acids, poly(ester amide)s, interfacial polymerization, scaffolds

## Abstract

Biodegradable poly(ester amide) (PEA) biomaterials derived from α-amino acids, diols, and diacids are promising materials for biomedical applications such as tissue engineering and drug delivery because of their optimized properties and susceptibility for either hydrolytic or enzymatic degradation. The objective of this work was to synthesize and characterize biodegradable PEAs based on the α-amino acids l-phenylalanine and l-methionine. Four different PEAs were prepared using 1,4-butanediol, 1,6-hexanediol, and sebacic acid by interfacial polymerization. High molecular weight PEAs with narrow polydispersity indices and excellent film-forming properties were obtained. The incubation of these PEAs in PBS and chymotrypsin indicated that the polymers are biodegradable. Human coronary artery smooth muscle cells were cultured on PEA films for 48 h and the results showed a well-spread morphology. Porous 3D scaffolds fabricated from these PEAs were found to have excellent porosities indicating the utility of these polymers for vascular tissue engineering.

## 1. Introduction

The development of biodegradable polymers is an increasingly important research area due to demands for such applications as sutures, surgical implants, controlled-release formulations for drugs, and scaffolds for tissue engineering [1,2,3]. Aliphatic polyesters are the most widely investigated family of biodegradable polymers. Although several variations of aliphatic polyesters based on glycolic acid, l-lactic acid, ε-caprolactone, and their copolymers have been used as biodegradable biomaterials [4,5,6], they still lack certain optimum properties [7,8]. For example, the degradation rate of aliphatic polyesters cannot be tuned easily as most of these polyesters are synthesized from single monomers. Attempts to tune the degradation rate often involves copolymerization or blending with other polymers that are not always successful [9]. In addition it has been shown that the acidic degradation byproducts of these polyesters to be toxic to some cells, limiting their use as functional tissue engineering scaffolds [10,11]. In an attempt to address these limitations, synthetic poly (α-amino acids) and polyamides containing α-amino acids have been considered [12,13,14], but they have not yet found the extent of expected applications, mainly because of preparation difficulties [15]. Recently, α-amino acid-based poly(ester amide)s (PEAs) have been proposed and investigated as a potential family of biodegradable polymers with good processing properties, and susceptibility to both hydrolytic and enzymatic degradation [16]. The likely low toxicity of their degradation products due to their natural origin and the fact that amino acids can be targeted for cleavage by proteolytic enzymes are important benefits of PEAs derived from α-amino acids. One of the earliest attempts to synthesize biodegradable PEAs based on α-amino acids was reported by Saotome *et al.* [17,18]. Despite this novel approach, the polymers were insoluble in many solvents to be fully characterized. Following this early attempt, a systematic study on the synthesis and characterization of PEAs derived from the amino acids glycine and l- and l,d-alanine has been conducted by Puiggali and coworkers [12,19,20,21,22,23,24,25]. These authors used an interfacial polycondensation to obtain highly crystalline PEAs with the number average molecular weight up to 10,000. Due to the low molecular weights of the polymers synthesized from glycine, it was not, however, possible to obtain films limiting the practical use of these polymers [19]. The polymers based on l-alanine, on the other hand, had film forming capabilities when selected diacids and diols are used as comonomers, but detailed characterization of the polymers including the molecular weights were not reported [20]. Preliminary biodegradation studies demonstrated that these polymers degrade both hydrolytically and enzymatically [23]. The enzymatic degradation of the PEAs with papain was stereospecific and appreciable differences in the degradation rate were found between the stereoregular and the racemic polymers, indicating that papain is more effective where the natural l-amino acid is involved [23]. In general, it is to be expected that derivatives of glycine and l-alanine to be highly crystalline with extensive hydrogen bonding in contrast to the amorphous character of polymers that could be synthesized from α-amino acids with bulky side groups [15].

The group of Katsarava and Chu [26] reported the synthesis and characterization of an interesting family of PEAs from di-*p*-toluenesulfonic acid salts of bis(α-amino acid)- α,ω-alkylene diesters and di-*p*-nitrophenyl esters of diacids by solution polycondensation. In this study, film forming PEAs having a range of molecular weights with narrow polydispersities were reported. The potential use of some of these PEAs for the controlled release of drugs and genes has been recently reported [27,28,29]. Kise and coworkers [30] also reported PEAs prepared under similar conditions, but the polymers had generally lower molecular weights. Among all the PEAs that were synthesized by this group, those based on l-phenylalanine showed the highest tendency toward the α-chymotrypsin-catalyzed hydrolysis, while the hydrolysis of the d, l-isomer occurred at a lower rate than the l-isomer. The high hydrophobicity of the benzyl side groups in the phenylalanine-based PEAs was suggested to be responsible for the enhanced enzymatic hydrolysis.

Despite the aforementioned studies on the synthesis of different PEAs based on α-amino acids, there is little evidence that these polymers could be used as useful biomaterials for tissue engineering applications [31]. We, therefore, hypothesize that PEAs derived from α-amino acids have a significant potential as scaffolding materials for tissue engineering applications as they could be degraded either by simple hydrolysis or by the action of proteolytic enzymes. It is also possible that the acidic and basic degradation products of these PEAs to have a buffering effect and, could potentially enhance cell-material interactions. This is particularly important since current degradable polyesters are known to release acidic products that adversely affect cellular behavior in long culture times [5]. In this study, we report the syntheses and biodegradation of versatile PEAs derived from l-phenylalanine and l-methionine. Furthermore we showed the attachment and spreading of human coronary artery smooth muscle cells (HCASMCs) on these PEAs. Finally, using these PEAs, we fabricated porous scaffolds demonstrating their suitability for vascular tissue engineering applications.

## 2. Results and Discussion

### 2.1. Polymer synthesis and characterization

**Scheme 1:**
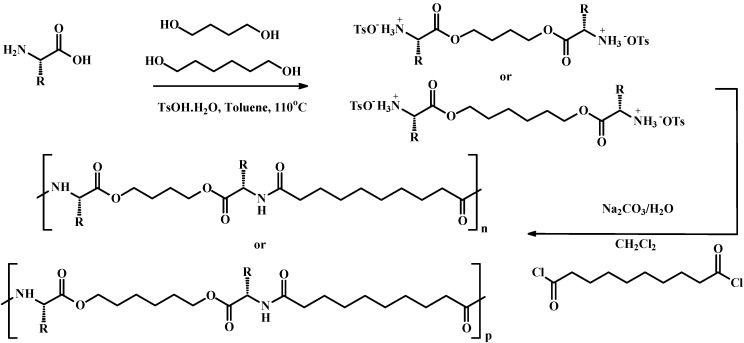
Synthetic pathways for preparing PEAs from the α-amino acids l-phenylyalanine and l-methionine.

The current PEAs were prepared from l-phenylalanine, l-methionine, 1,4-butanediol, 1,6-hexanediol and sebacoyl chloride by interfacial polymerization according to Scheme 1. l-phenylalanine was chosen due to its aromatic ring that will give rigidity to the PEAs and due to its propensity for enzymatic hydrolysis. l-methionine was chosen because it has an aliphatic side chain. Thus the use of these two amino acids would allow the synthesis of both aromatic and aliphatic side chain PEAs that are largely amorphous. The use of l-methionine in this work is the first report because only racemic mixture has been reported in the literature.

**Figure 1:**
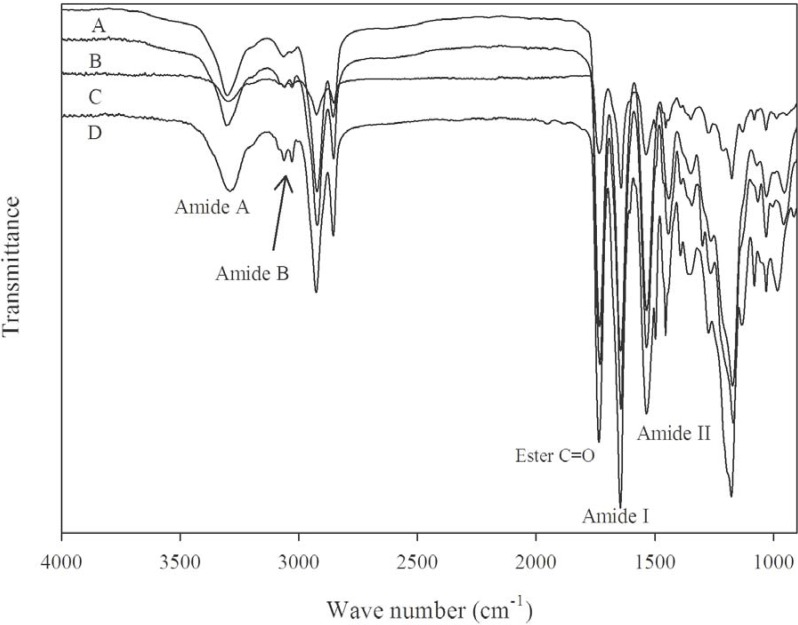
FTIR Spectra of PEAs synthesized from l-phenylalanine and l-methionine. (A) PEA-Ph48; (B) PEA-Ph68; (C) PEA-Me48; and (D) PEA-Me68.

The structures of the synthesized PEAs were confirmed by FTIR and ^1^H-NMR spectra. The FTIR spectra in Figure 1 showed strong primary amide absorption band at around 3300 cm^-1^ (amide A), a peak at around 3050 cm^-1^ for the intermolecular hydrogen bonded amide (Amide B), 1680 cm^-1^ symmetric N-C bond (amide I) and 1520 cm^-1^ asymmetric N-C bond (amide II). As can be seen, the amide I region is more intense than amide II indicating symmetry in the structures of the PEAs. The peak at 1740 cm^-1^ corresponds to the ester carbonyl (C = O).

**Figure 2:**
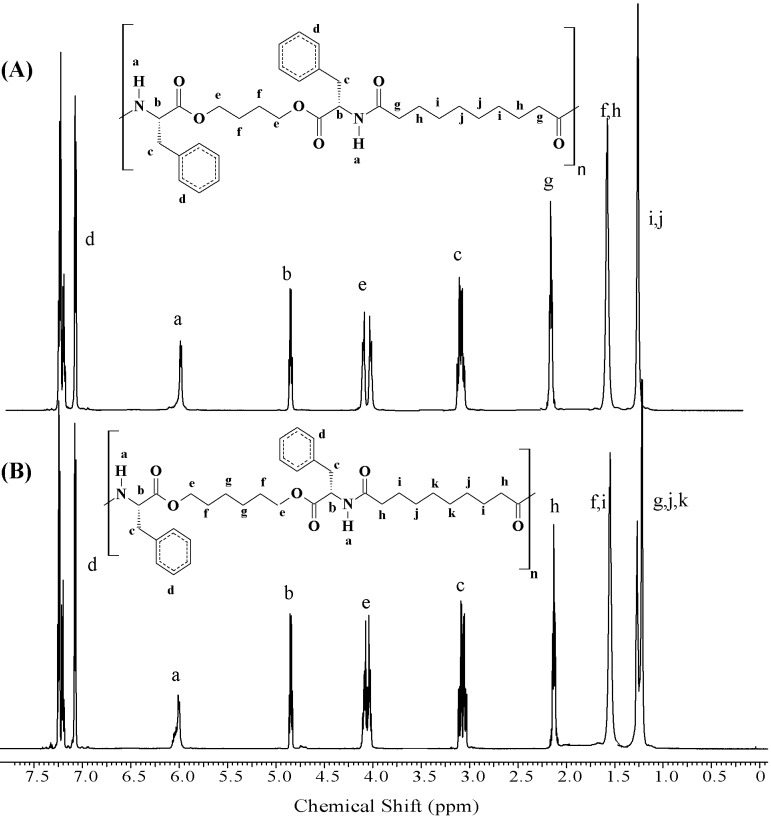
^1^H**-**NMR spectra for PEAs synthesized from l-phenylalanine. (A) PEA-Ph48 and (B) PEA-Ph68.

**Figure 3:**
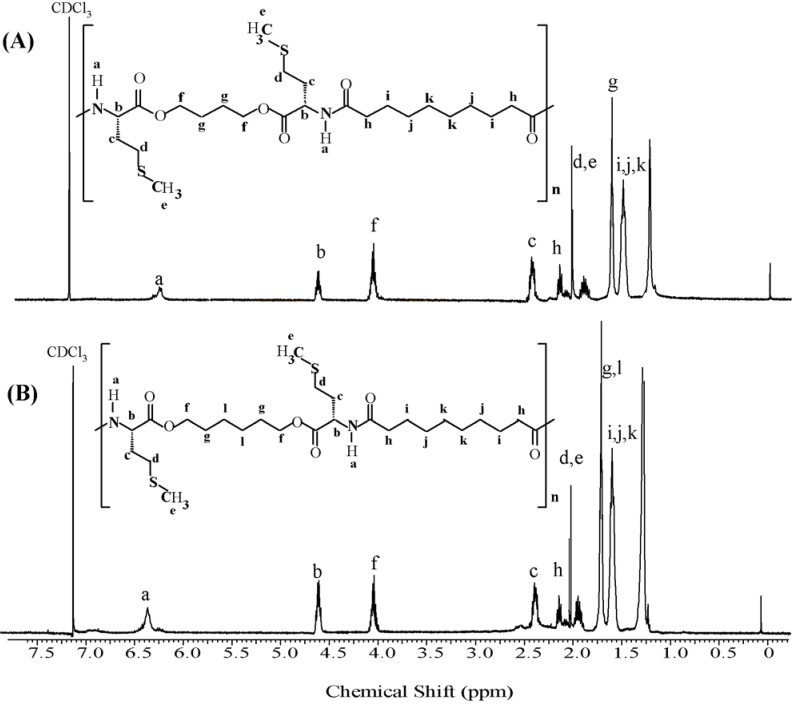
^1^H**-**NMR spectra for PEAs synthesized from l-methionine. (A) PEA-Me48 and (B) PEA-Me68.

The ^1^H-NMR spectra of the PEAs are shown in Figure 2 and Figure 3, from which it can be seen that the polymers were obtained free from impurities and any detectable end groups. All the assigned peaks are those expected from the polymer structures.

**Figure 4:**
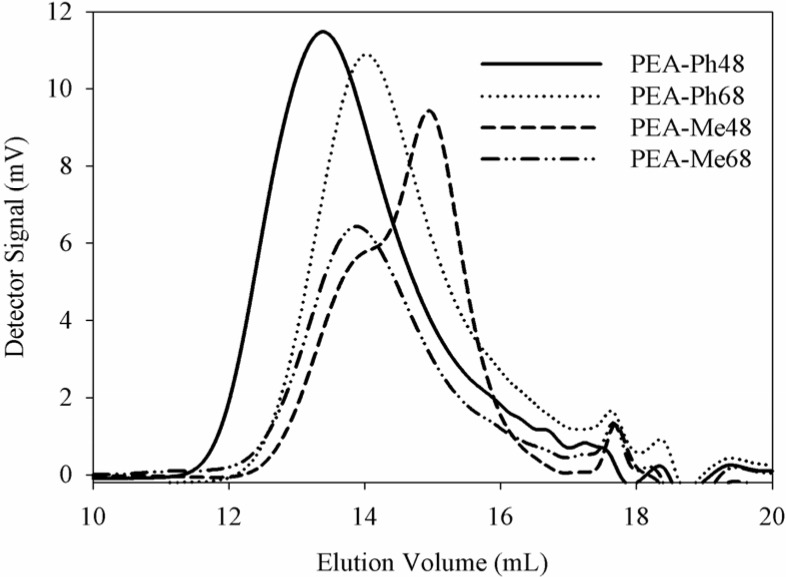
GPC traces of PEAs synthesized from the amino acids l-phenylalanine and l-methionine.

In this work, we chose interfacial polymerization instead of solution polymerization for the following reasons. Firstly, low temperature interfacial polymerization is attractive since high rates of reaction outpace any competing side reactions thus allowing the synthesis of linear polymers possessing high molecular weights. Thus monomer molecules will react with the growing polymer chain ends before they can penetrate through the polymer film to start the growth of new chains. This, in turn, means that the polymerization times can be significantly reduced. For instance, when PEAs were prepared by solution polymerization, reaction times in excess of 48 h were needed to obtain any useful polymer products [26,33]. As we described in our experimental section, the reaction time we used was a maximum of 2 h. Secondly, the restrictions on purity and stoichiometry are less stringent in interfacial polymerization than those for solution polymerization and a common solvent for the monomers and the polymer is not necessary. Therefore, the formation of high molecular weight polymers at the interface is easily achieved regardless of the overall percent conversion based on the bulk amounts of the two reactants [34]. This is in sharp contrast with solution polymerization which requires strict stoichiometry and high purity of monomers. As can be seen from our molecular weight data (Table 1 and Figure 4), the number average molecular weight of the polymers were in the range of 28,000 to 45,000 g/mol with polydispersity indices (PDI) in the range of 1.32 to 1.56. PEA-Ph48 had notably higher molecular weight and PDI than the other PEAs. This was unexpected as the polymerization method was the same in all PEAs and we anticipated PEA-Ph48 to have similar molecular weights with PEA-Ph68. In order to rule out experimental errors, multiple molecular weight measurements were conducted but consistent results were obtained. The effect of the phenyl side chain that can form an extended conformation (and hence large hydrodynamic volume) leading to high molecular weight is not a plausible explanation because, had this been the case, PEA-Ph68 with the same side chain should have similar molecular weight. It is therefore conceivable that the apparent high molecular weight for PEA-Ph48 to be the result of other factors that are not known at this stage. Despite this difference, all PEAs had high molecular weights to prepare films for degradation studies and cell culture, and to fabricate scaffolds that could be used for tissue engineering applications. The relatively low PDI for these PEAs is also interesting since it is generally known that the molecular weight distributions observed in interfacial polymerizations are usually broader than the most probable distribution [35]. The differences are probably due to the solvent used as an organic phase during the polymerization that leads to fractionation when the polymer undergoes precipitation. This effect essentially allows only the high molecular weight polymers to precipitate thus forming a narrow molecular weight distribution. We should also mention that the molecular weights of most PEAs reported in the literature [19,33,36] are much lower than the ones we obtained. Sufficiently higher molecular weight PEAs that are capable of film and fiber forming are needed for practical applications such as drug delivery and scaffolds for tissue engineering.

**Table 1:** Yields, molecular weights, and glass transition temperatures of PEAs synthesized from l-phenylalanine and l-methionine.

Polymer	Sample Name	Yield (%)	*M_n_* (g/mol)	*M_w_* (g/mol)	PDI	*T*_g_ (°C)
l-phenylalanine, 1,4-butanediol, sebacoyl chloride	PEA-Ph48	85.15	45,070	70,596	1.56	39
l-phenylalanine, 1,6-hexanediol, sebacoyl chloride	PEA-Ph68	81.03	32,538	45,008	1.38	28
l-methionine,1,4-butanediol, sebacoyl chloride	PEA-Me48	63.21	27,974	37,050	1.32	-2
l-methionine, 1,6-hexanediol, sebacoyl chloride	PEA-Me68	78.09	35,913	49,481	1.38	-1

Finally, the current PEAs were obtained in good yields and free from any residual byproducts compared to those prepared by solution polymerization [37]. After washing with acetone and ethanol, the yields for the current PEAs based on l-phenylalanine were generally higher than those based on l-methionine. The lower yields for PEA-Me48 and PEA-Me68 compared with PEA-Ph48 and PEA-Ph68 are attributed to the improved solubility in purification solvents due to the aliphatic nature of the amino acid used. In the past, one of the major challenges in synthesizing PEAs from α-amino acids using interfacial polymerization was the low yield and/or low molecular weight of the resulting polymers. For instance PEAs based on l-alanine have been reported to give reasonable yield but the molecular weight was below 10,000 g/mol whereas, those based on glycine had a poor yield [19,20]. PEAs prepared from solution polymerization are also known to have poor yields [26,30,37]. By carefully selecting the l-amino acids and the diols, we synthesized PEAs with high yields that can easily be scaled up to prepare large quantities. For biomedical applications, the purity of the polymer is as important as its molecular weight. The solution polymerization method to prepare PEAs from α-amino acids invariably utilizes *p*-nitrophenol to form the diester of the carboxylic acid [26,33,37]. Removal of *p*-nitrophenol, which is a known cytotoxic chemical [38], following the polymerization is laborious [26]. Because of the polymerization method we employed, such a cytotoxic chemical was not needed and the PEAs obtained were of high purity that could be suitable for cell culture (see later section).

### 2.2. Thermal analysis

**Figure 5:**
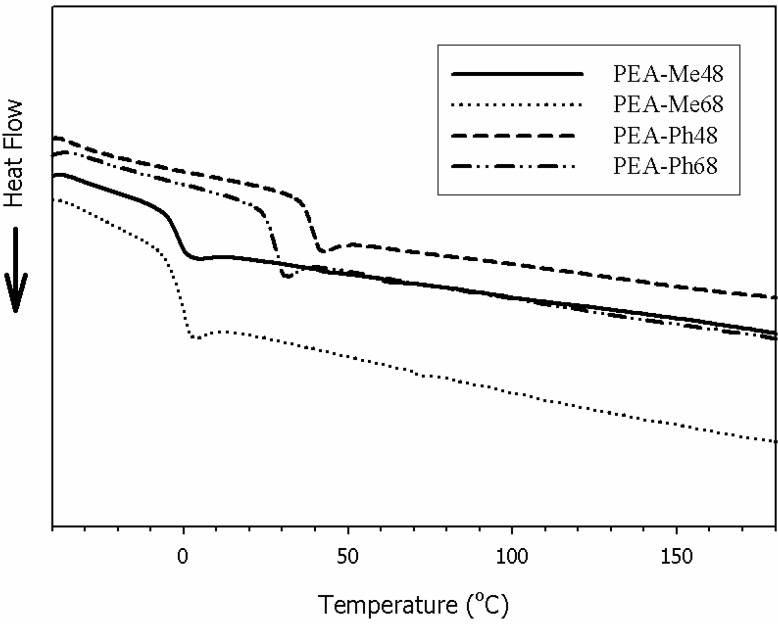
DSC thermograms of PEAs synthesized from the amino acids l-phenylalanine and l-methionine.

Figure 5 shows the DSC traces of the PEAs from −50 °C to 200 °C. As can be seen, the l-methionine-based PEAs had *T*_g_s around 0 °C whereas those PEAs derived from l-phenylalanine had higher Tgs. It is also interesting to note that the change of the diol from butanediol to hexanediol in the l-phenylalanine PEAs had a noticeable *T*_g_ difference (39 °C *vs.* 28 °C). However, the different diols had no effect on the *T*_g_ of PEAs based on l-methionine. This is probably due to the flexible side chains of the amino acid l-methionine that could effectively dominate the transition temperature such that the effect of the diol chain length is not appreciable. None of the PEAs showed a melting and crystallization peak during the second heating cycle indicating that the polymers were amorphous. This is to be expected since both l-phenylalanine and l-methionine side chains can inhibit close packing and eliminate crystallization. An x-ray diffraction study (data not shown) also indicated the absence of crystallization.

**Figure 6:**
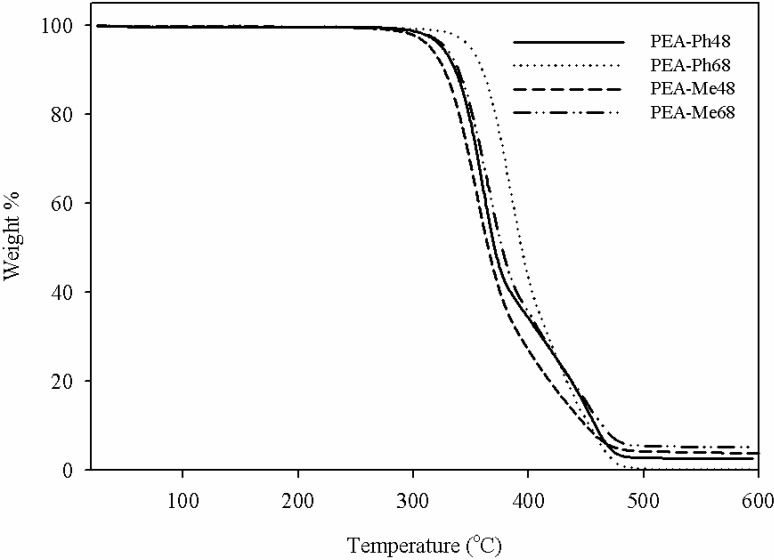
Thermogravimetric analysis of PEAs synthesized from the amino acids l-phenylalanine and l-methionine.

Thermogravimetric analyses of the current PEAs are shown in Figure 6. It can be seen that PEA-Me48 was the least thermally stable due to its aliphatic chains and the cleavage of the carbon-sulfur bond which is known to be a weak link [39], whereas PEA-Ph68 is the most stable owing to its aromatic side chain. With the exception of PEA-Ph68, the onset degradation temperature for all the PEAs was ~280 °C. Taken together, the DSC and TGA data demonstrated that the current PEAs can be melt processed to fabricate scaffolds for tissue engineering applications or other devices without degradation.

### 2.3. Biodegradation studies

**Figure 7:**
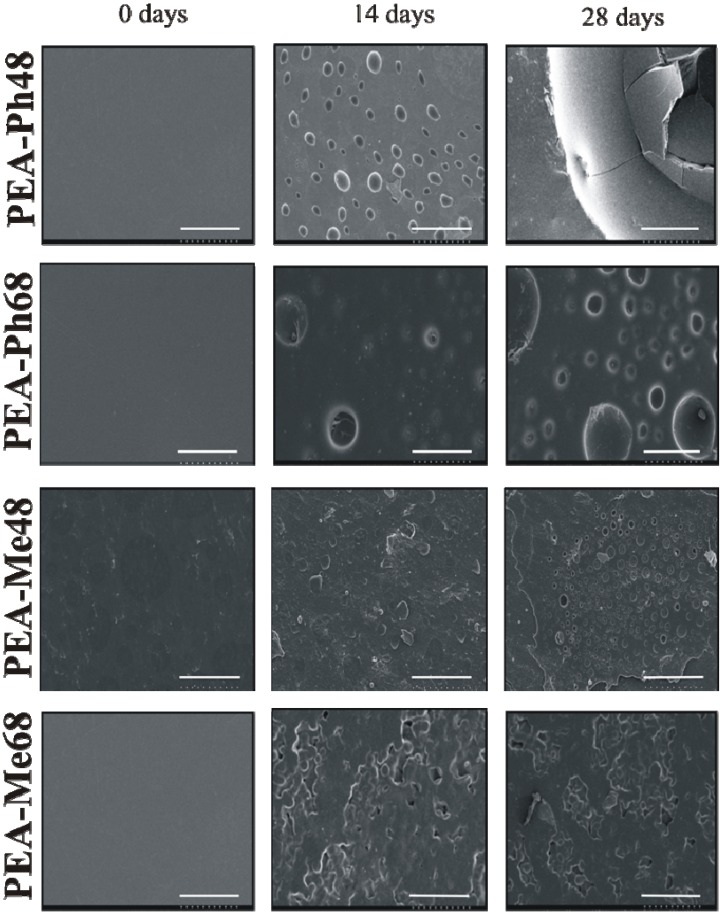
SEM images of PEA films degraded in PBS at 37 °C for up to 28 days. At day 28, PEA-Ph48 and PEA-Ph68 cracked or blistered wheras PEA-Me48 and PEA-Me68 eroded. Scale bar = 100 μm.

**Figure 8:**
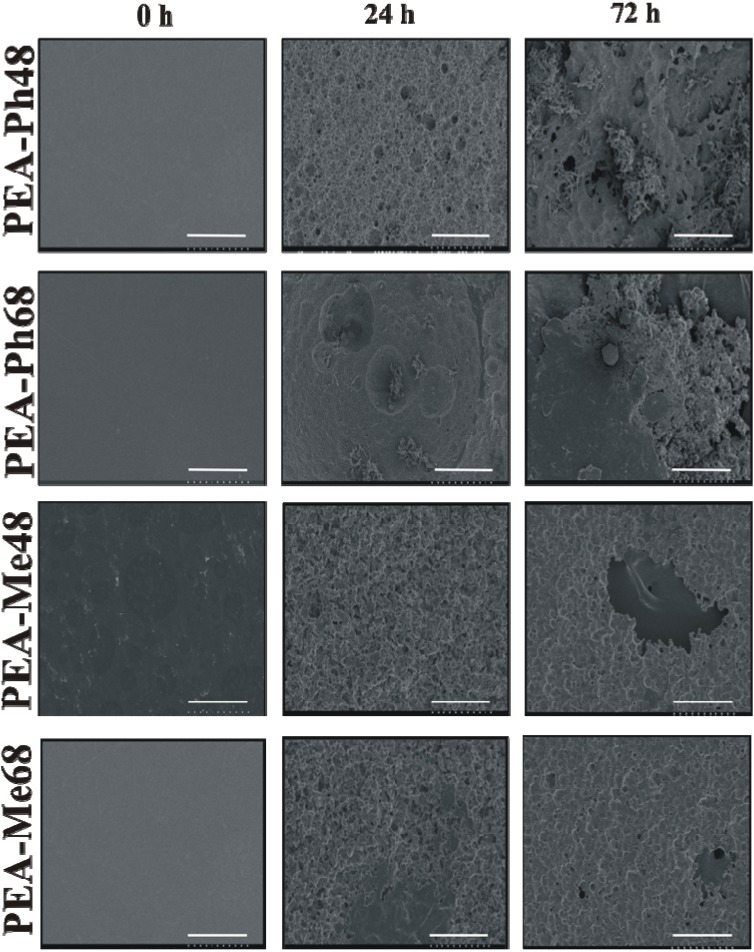
SEM images of PEA films following 72 h of incubation in chymotrypsin at 37 °C. All polymers showed an extensive erosion but the l-phenylalanine PEAs are the most eroded. Scale bar = 100 μm.

Biodegradation studies were performed on thin films of PEAs to evaluate the hydrolytic and enzymatic degradation. Chymotrypsin was selected due to its preference to hydrophobic amino acids while it is also known to cleave the C-terminal bond of aromatic amino acids in various peptides under physiological conditions [17,40]. Following incubation, films were imaged by SEM which is an ideal technique for investigating topological changes due to degradation of these polymers. The SEM images of the PEA films before and after degradation in PBS are shown is Figure 7. Prior to incubation (day 0), all films were smooth. After 14 days of incubation, small blister formation on all the films was evident, but it was more pronounced for the l-methionine-based PEAs. At day 28, the l-phenylalanine-based PEAs either cracked or blistered and the surface appeared eroded; whereas, the l-methionine-based PEAs have shown significant degradation. This difference probably results from the increased hydrophilicity of PEA-Me48 and PEA-Me68 that could allow increased water penetration into these films. The SEM images of films before and after incubation in chymotrypsin are presented in Figure 8. It is clear that the presence of chymotrypsin accelerated the degradation within 72 h particularly for PEA-Ph48 and PEA-Ph68. The rapid degradation of PEA-Ph48 and PEA-Ph68 under enzymatic conditions may be explained by the preference of chymotrypsin for hydrophobic amino acids and also by the high level of hydrolytic degradation, which leads to a rapid increase in polymer surface area, allowing the enzyme to readily access the polymer [41]. Beyond 72 h, the integrity of the degraded films was compromised to withstand the SEM imaging procedures and the data were discarded.

**Figure 9:**
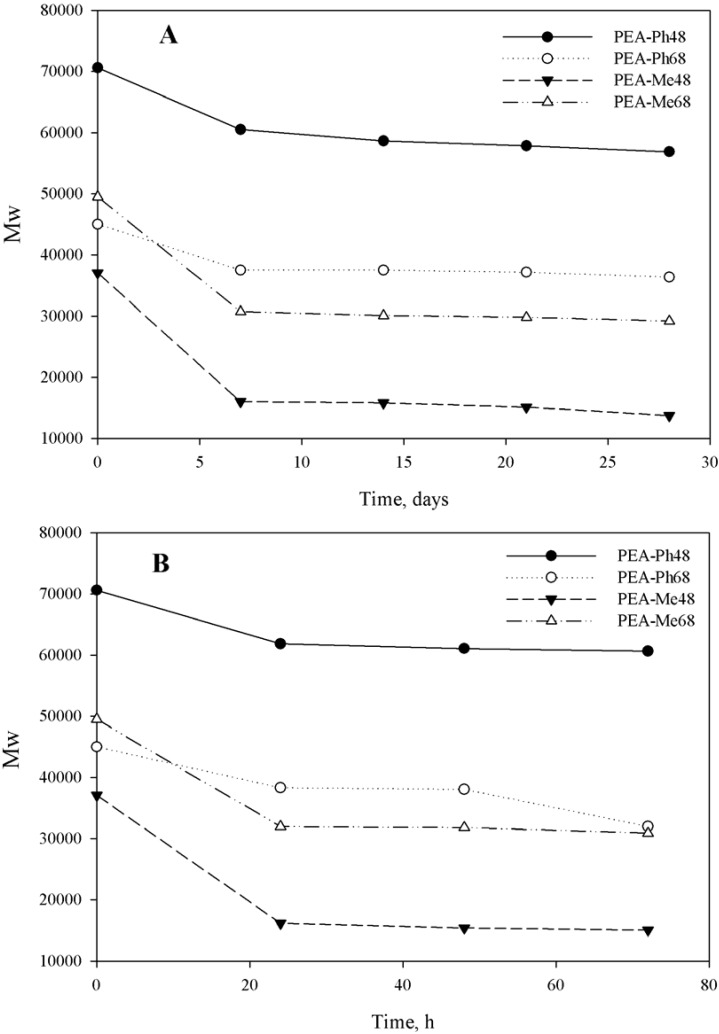
Molecular weight changes of PEAs incubated in (A) PBS at 37 °C for up to 28 days and, (B) chymprtypsin at 37 °C for up to 72 h.

SEM is a qualitative characterization method that provides an insight on surface properties, but it provides little information in terms of the properties of the bulk polymer as it degrades. The molecular weight changes, on the other hand, could provide additional information regarding the possible degradation mechanisms. For example, according to SEM, PEA films may appear to be degraded on the surface but the rest of the bulk material may not be affected. Initially we have verified that the mass of the degrading polymers decreased with time (data not shown). However, mass loss is a necessary but not a sufficient condition for polymers undergoing surface erosion because bulk degradation is also accompanied by mass loss. For this purpose, molecular weights as a function of degradation time in PBS and in chymotrypsin were measured. Figure 9 shows the molecular weight changes over the degradation times investigated.

From Figure 9, three points can be made. First, the polymers degraded in PBS for 28 days and those degraded in chymotrypsin for 72 h had similar molecular weights indicating that chymotrypsin indeed accelerated the degradation. This is consistent with our SEM observations. Second, there is a step initial decrease of the molecular weight for all PEAs followed by an almost no change for the rest of the experimental degradation times both in PBS and in chymotrypsin. This suggests that the current PEAs are undergoing an initial bulk degradation followed by surface erosion for the remainder degradation times studied. Obviously, surface erosion reduces the weight of the polymer samples but should not affect the molecular weight of the remaining materials significantly. Third, the mechanism of degradation seems to be unchanged by the presence of chymotrypsin as the molecular weight trends are very similar to that of PBS, despite the rate of degradation. Taken together, our molecular weight data suggested that the degradation of PEAs is less dependent on the type of amino-acids. Many studies in the past focused only on weight loss measurements to gain an understanding on the degradability of PEAs [23,30,33,42]. We believe that for biodegradable polymers intended as medical devices and tissue engineering scaffolds, weight loss alone is not sufficient to understand mechanism of degradation and determine the practical application. For example, a polymer could lose its weight, but as long as the molecular weight of the remaining polymer is high enough, it can still possess sufficient properties.

### 2.4. Vascular smooth muscle cells interaction with PEAs

**Figure 10:**
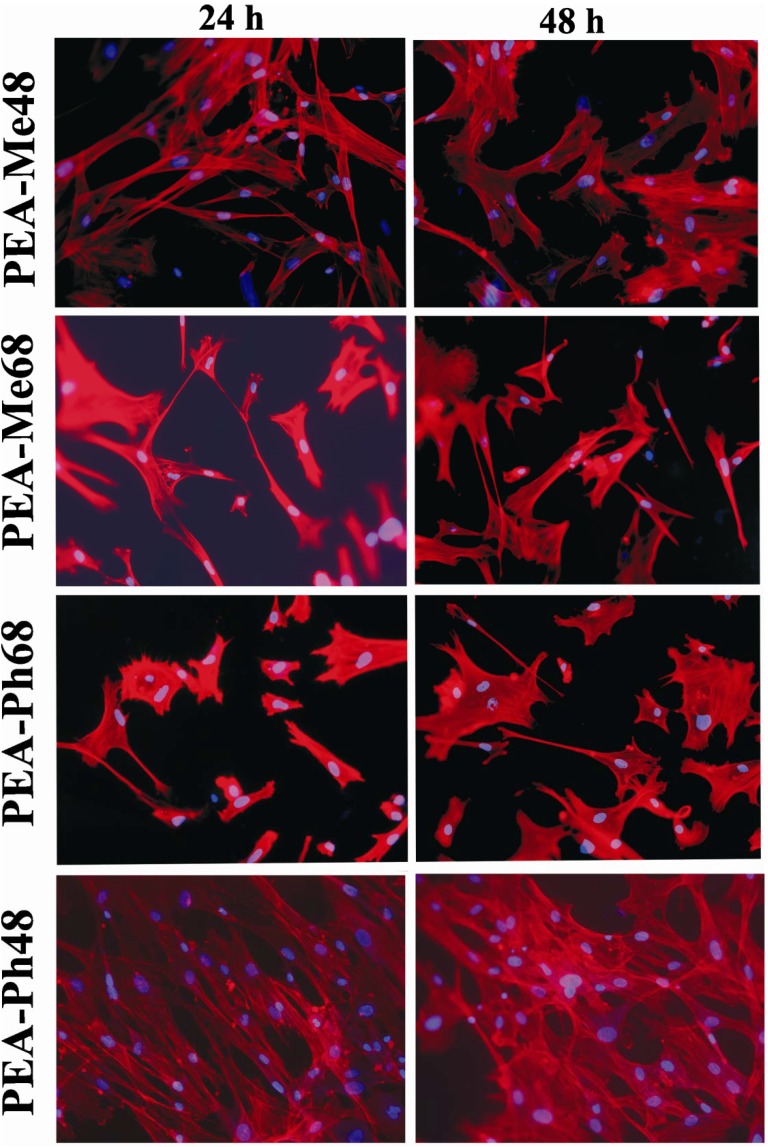
Human coronary artery smooth muscle cells attachment and spreading on films of PEAs synthesized from the amino acids l-phenylalanine and l-methionine. A well-spread morphology is evident for all the polymers and up to 48 h culture time.

In order to evaluate to what extent the current PEA surfaces support the attachment and spreading of cells, HCASMCs were seeded and cultured for up to 48 h. Labeling for F-actin and DNA were used to examine cellular morphology. As presented in Figure 10, cells behaved in a similar fashion regardless of the polymer types used. After 24 h of culture, well-spread and irregularly shaped individual HCASMCs were encountered that contained prominent F-actin bundles. Following 48 h in culture, cells remained sparse but were elongated and formed both cell-cell and cell-material adhesion. There are early indications that cells are beginning to form aligned aggregates with increased actin stress fibers. We did not observe any evidence of increased cell numbers but given that the culture time was short, only cell adhesion and spreading were evident on the current PEAs. Since cell adhesion and spreading are the first events that dictate the subsequent cellular responses such as proliferation, migration and matrix deposition, it is important that biomaterials are designed to enhance these initial events. Clearly, the current PEAs are demonstrated to promote adhesion and spreading of HCASMCs.

Whereas the use of PEAs derived from non-amino acid precursors have been studied as cell supporting biomaterials [11,43,44,45] the attachment and spreading behavior of cells on PEAs derived from α−amino acids is notably absent from the literature. In fact our study is only preceded by two previous studies on glycine [31] and l-alanine [20] containing PEAs where it was shown both PEAs to be less favorable to cells. In the extreme case of glycine, fibroblast cells did not exhibit spreading and remained spherical in appearance and the growth rate was significantly reduced [31]. The favorable vascular smooth muscle cell attachment and spreading results found in our study is very likely to be due to the absence of ordered structure in the PEAs we have prepared. As shown in the DSC data, the current PEAs were non-crystalline and the absence of ordered structures may have presented a favorable surface on the film such that serum proteins from the culture media is preferentially adsorbed and provided the necessary cellular cue for attachment and spreading.

### 2.5. Scaffold fabrication

**Figure 11:**
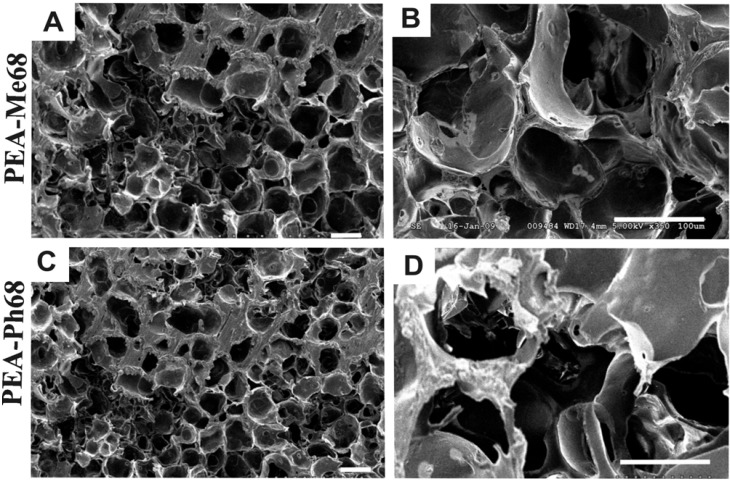
Porous 3D scaffolds fabricated from PEAs. Scale bar = 100 μm.

To evaluate the possibility of the current PEAs as potential scaffolds for tissue engineering applications, we fabricated 3D scaffolds. The results presented in Figure 11 show that some of these polymers are suitable candidates. Scaffolds made of PEA-Ph48 and PEA-Me48 were too weak and could not be cut for SEM images. On the other hand, as it can be seen in Figure 11, scaffolds fabricated by PEA-Ph68 and PEA-Me68 were highly porous with interconnected pores suggesting the potential utility of these PEAs for tissue engineering applications.

Intuitively one of the main challenges in the tissue engineering is the need for a suitable supporting scaffold for the control of cell behavior and organization. Scaffolds should have suitable surface chemistry to promote cellular attachment, migration, and proliferation. It should also exhibit appropriate pore size and high pore interconnectivity to promote cell infiltration, and degradation products must not be cytotoxic. Our preliminary studies indicate that the current PEAs met most of the required scaffold properties. This is the first time that porous scaffolds are fabricated from PEAs derived from amino acids and, this opens an opportunity to further explore these PEAs as the next generation scaffold materials.

## 3. Experimental Section

### 3.1. Materials and methods

All chemicals were purchased from Sigma Aldrich (Milwaukee, WI, USA) and were used without further purification. The α-amino acids used in this study were l-phenyalanine and l-methionine. For the enzymatic degradation studies, α-chymotrypsin from bovine pancreas type II, 83.9 units/mg was used.

### 3.2. Synthesis of l-phenylalanine/l-methionine, and 1,4-butanediol/1,6-hexanediol diester p-toluene sulfonate.

Similar to a previously reported procedure in the literature [19], a 3-neck, round bottom flask equipped with a Dean–Stark apparatus, and a magnetic stirrer was charged with diol (27.5 mmol), α-amino acids (60.5 mmol), p-toluenesulfonic acid (66 mmol), and toluene (125 mL). The reaction mixture was refluxed for 10 h until no more water was produced. Then, it was cooled to room temperature and the toluene evaporated under reduced pressure (0.013 kPa) for 24 h. The resulting l-phenylyalanine-based diesters were recrystallized from water while the l-methionine-based diesters were recrystallized from isopropanol. Yield 85–91%.

^1^H NMR (400 MHz, D_2_O): l-phenylalanine/1,4-butanediol and l-phenylalanine/1,6-hexanediol diesters: δ 7.70–7.20 (Ar-**H**), 4.25 (NH_3_^+^C**H**COO-), 4.10 (-COOC**H_2_**-), 3.20 (C**H_2_**-Ar), 2.25 (C**H_3_**-Ar), 1.65 (-COOCH2C**H_2_**-), 1.20 (-C**H_2_**-). mp: 235 °C, 191 °C. l-methionine/1,4-butanediol and l-methionine/1,6-hexanediol diesters: δ 7.2–7.7(Ar-**H**), 4.55 (-OOC**H_2_**-), 4.30 (NH_3_^+^C**H**COO-), 2.48(Ar-C**H_3_**), 2.24(-C**H_2_**S-), 1.95(-SC**H_3_**), 1.65(-C**H_2_**-). mp: 205 °C, 162 °C. IR ν_max_/cm^-1^ for all diesters: 3050–3190 (Ar), 2910 and 2845 (CH_2_), 1750(C = O), 1190 and 1130 (CO-O).

### 3.3. Synthesis of PEAs by interfacial polymerization (Scheme 1)

Dichloromethane was used as the organic phase, and sodium carbonate (Na_2_CO_3_), as a proton acceptor. A water solution (45 mL) of the diester (15 mmol; 1 eq) and Na_2_CO_3_ (30 mmol; 2 eq) was added dropwise into a stirred solution of sebacoyl chloride (15 mmol; 1 eq)) in dry dichloromethane (40 mL). After the addition was completed, stirring was continued for 2 h. The polymer, which precipitated, was isolated by filtration and successively washed with water, ethanol, and acetone before drying in a vacuum desiccator at 60 °C. Accordingly a total of four different PEAs were synthesized: Yield 63–85%. The PEAs are named according to the first two letters of the amino acids and the number of carbon atoms in the diols used to synthesize them. Thus, PEA-Ph48 means a poly(ester amide) synthesized from l-phenylalanine and 1,4-butanediol. The number 8 which is common to all polymers indicates sebacoyl chloride.

### 3.4. Spectroscopic analyses

Fourier Transform Infrared (FTIR) spectra were recorded by averaging 32 scans at a resolution of 1/cm using Bruker Vector 22 spectrophotometer. Nuclear Magnetic Resonance (^1^H-NMR) spectra were obtained on a Varian^®^ INOVA 400 instrument operating at 400 MHz. Chemical shifts are reported in ppm and were calibrated against residual solvent signals of CDCl_3_ (δ 7.26 ). D_2_O was used for the diesters and CDCl_3_ for the PEAs.

### 3.5. Gel permeation chromatography (GPC)

The molecular weights of the PEAs before and after degradation were determined in N,N-dimethylformamide (DMF) with 10 mM lithium bromide (LiBr) and 1% (v/v) triethylamine (TEA) at 85 °C and a flow rate of 1 mL/min. The GPC system included a Waters 2695 separations module equipped with a Waters 2414 differential refractometer and two PLgel 5 µm mixed-D (300 ° 7.5 mm) columns connected in series (Polymer Laboratories, Varian Inc., MA, USA). Samples were calibrated with polystyrene standards. All data were collected and processed using Waters Empower II Software.

### 3.6. Differential scanning calorimetry (DSC)

DSC scans were obtained on a DSC Q200 V24.2 Build 107 (TA Instruments, New Castle, DE). The samples (ranged from 10–20 mg) were placed in aluminum pans, hermetically sealed, and heated from −50 °C to 200 °C at a rate of 10 °C/min while purging nitrogen at rate of 50 mL/min. Glass transition temperatures (*T*_g_s) were obtained from the second heating cycle.

### 3.7. Thermogravimetric analysis (TGA)

TGA was carried out using a Q-series TGA Q 500 analyzer (TA instruments, New Castle, DE). The specimens, dried overnight at 60 °C in a vacuum oven, were weighed in the range of 5 to 10 mg and heated from 25 °C to 600 °C at a heating rate of 10 °C/min under nitrogen. TA Instruments Universal Analysis 2000 software was used to analyze the data.

### 3.8. Biodegradation study

Films for PEA-Ph48 and PEA-Ph68 were obtained by heat pressing the polymers at 150 °C and 200 kPa for 2 min. Films for PEA-Me48 and PEA-Me68 were obtained by dissolving the samples in chloroform at a concentration of 50 mg/mL followed by casting in a circular Teflon dish. The solvent was allowed to evaporate followed by drying under vacuum at 50 °C for 12 h. Film specimens of 6 mm diameter and ~0.11 mm thickness were punched and incubated at 37 °C in vials containing either 15 mL phosphate buffered saline (PBS; pH 7.4) for up to 28 days, or PBS containing α-chymotrypsin (0.5 mg/mL) for up to 72 h. The films were removed from the solution, rinsed with distilled water and dried for further analyses.

### 3.9. Sample preparation for cell culture

PEA films were punched into small discs with a diameter of 1.2 cm to fit 24-well cell culture plates, affixed to coverslips using silicone grease and sterilized by immersion in 70% ethanol for 30 min. Following this, the films were washed with sterile PBS and transferred to 24-well cell culture plates (BD Biosciences, San Jose, CA) and incubated in 1 mL of culture medium at 37 °C overnight before cell seeding for preconditioning.

### 3.10. Cell culture and immunofluorescence microscopy

Human coronary artery smooth muscle cells (HCASMCs; Cambrex Biosciences, Walkersville, MD) between passage 6 and 10 were used to evaluate the attachment and spreading of vascular cells on PEA films. HCASMCs were cultured in SmGM-2 (smooth muscle growth medium-2 with Bullet Kit; Cambrex) supplemented with 1% penicillin/streptomycin solution (P/S). Cultures were maintained in a humidified incubator at 37 °C and 5% CO_2_. HCASMCs were seeded onto films at an initial cell density of ~10^4^ cells/cm^2^ and cultured for 24 and 48 h before fixation and immunostaining. Following culture time, cells were fixed at 4 °C for overnight in 4% paraformaldehyde (EMD Chemicals, Gibbstown, NJ) in divalent cation-free phosphate-buffered saline (PBS) and permeabilized with 0.5% Triton X-100 in PBS. HCASMCs were incubated for 1 h at ambient temperature in 1% BSA/PBS containing Alexa Fluor® 568 phalloidin (Invitrogen, Eugene, OR; 1:50 dilution). Hoechst 33342 (10 µg/mL; Sigma-Aldrich) dissolved in PBS was used to label nuclei. Coverslips were mounted on glass slides in Vectashield (Vector Laboratories, Burlington, ON) and sealed with nail enamel. Samples were analyzed with a Leica DM-IRB fluorescence microscope equipped with epifluorescence and the appropriate filters (Leica, Canada).

### 3.11. Scaffold fabrication

Scaffolds were fabricated using a combination of pressure differential and particulate leaching techniques described in our previous publication [32]. Briefly, 1.80 g of ground and sieved ammonium chloride (NH_4_Cl) particles (180–250 µm) were packed into a cylindrical infiltration chamber, and the polymer solution (0.5 g dissolved in 2 mL chloroform) was subsequently poured over the porogen bed. The solution was allowed to infiltrate through the porogen assembly using a pressure differential. Following infiltration, the constructs were removed from the assembly and, the solvent was allowed to evaporate for at least a day in a fume hood. The porogen was then leached out using deionized water.

### 3.12. Scanning electron microscopy (SEM)

Dry PEA films (before and after degradation) and porous scaffolds were mounted on aluminum stubs with carbon tape, then sputter coated with a thin layer of gold. The surface microstructure of films and pore geometry of scaffolds were imaged using SEM (S-2600N, Hitachi, Japan).

## 4. Conclusions

Four new PEAs derived from the amino acids l-phenylalanine and l-methionine were successfully prepared by interfacial polymerization in high yields and adequate molecular weights to give film-forming materials. The PEAs were biodegradable and the degradation process can be accelerated using the enzyme chymotrypsin. Our degradation studies suggest that the current PEAs were undergoing an initial bulk degradation followed by surface erosion for the remainder degradation times studied. HCASMs were found to be well-spread on the current PEAs and; 3D porous scaffolds fabricated could have a potential to be useful candidates for tissue engineering applications.
